# Defining Epiglottic Collapses Patterns in Obstructive Sleep Apnea Patients: Francia-Lugo Classification

**DOI:** 10.3390/healthcare11212874

**Published:** 2023-10-31

**Authors:** Carlos Francia, Rodolfo Lugo, Antonio Moffa, Manuele Casale, Lucrezia Giorgi, Francesco Iafrati, Simone Di Giovanni, Peter Baptista

**Affiliations:** 1 Clínica Delgado, Lima 15074, Peru; cfrancia@auna.pe; 2 ISSSTE Constitución, Monterrey 64530, Mexico; 3 Integrated Therapies in Otolaryngology, Campus Bio-Medico University Hospital Foundation, 00128 Rome, Italy; 4 School of Medicine, Campus Bio-Medico University, 00128 Rome, Italy; 5 Unit of Measurements and Biomedical Instrumentation, Department of Engineering, Campus Bio-Medico University of Rome, 00128 Rome, Italy; 6 Department of Otorhinolaryngology, Clínica Universidad de Navarra, 31008 Pamplona, Spain; 7 ENT Department, Al Zahra Private Hospital Dubai, Dubai 23614, United Arab Emirates

**Keywords:** OSA, DISE, epiglottis collapse

## Abstract

Obstructive Sleep Apnea (OSA) is characterized by repetitive collapse of the upper airway during sleep. Drug-Induced Sleep endoscopy (DISE) is used to identify the collapse site. Among the possible sites of collapse, the epiglottis occurs more frequently than previously described. In this study, we reviewed DISE findings and classified different epiglottic collapse patterns. We found 104 patients (16.4%) with epiglottis collapse (primary 12.5% and secondary 3.9%). We described the following patterns of epiglottis collapse: Anterior–Posterior (AP) collapse with rigid component “trapdoor type” (48%); AP collapse with lax component “floppy type” (13.5%); Lateral– Lateral (LL) collapse with omega shape component “book type” (14.5%); and secondary due to lateral pharyngeal wall or tongue base collapse (24%). The identification of the epiglottic collapse pattern is crucial in decision-making when attempting to ameliorate OSA. These findings in OSA phenotyping could influence the type of treatment chosen.

## 1. Introduction

Obstructive Sleep Apnea (OSA) is the most prevalent chronic sleep-related breathing disorder [1,2,3]. It is characterized by apneas (complete) and/or hypopneas (partial) due to repetitive collapse of the upper airway during sleep, despite the effort to breathe [4]. The prevalence increases with age: between 30 to 70 years old adults is estimated to be 14% in males and 5% in females. In obese adults, the percentage ranges from 42% to 48% in men and 8% to 38% in women [5], and it is even higher in patients with cardiac or metabolic disorders [6]. OSA can be related to potential repercussions, such as excessive daytime sleepiness, impaired daytime function, exacerbation of metabolic abnormalities [7,8], and an increased risk of cardiovascular disease [9], chronic kidney disease, traffic accidents [10], and mortality [11]. Treatment with Continuous Positive Airway Pressure (CPAP) is the first-line treatment for OSA patients [12,13]. However, surgery may be indicated to improve compliance and outcomes in patients with poor tolerance to CPAP [14]. Moreover, it has been proven to reduce cardiovascular morbidity and mortality [15].

Airway obstruction in OSA patients are commonly multilevel and includes the Velopharynx, Oropharyngeal lateral walls, Tongue base, and Epiglottis (VOTE) classification [16]. Among the VOTE classification described by Kezirian, velopharyngeal obstruction is the most common type. Epiglottic collapse (EC) is the least common obstruction type and is usually ignored in the OSA surgical plan [17]. Although EC is usually ignored, the prevalence might be much higher.

In 1998, Catalfumo et al. [18] estimated that epiglottis collapse occurs in 11.5% of adult OSA patients using awake endoscopy as a diagnostic technique. Lan et al. [19] have found that it occurs more frequently and that 42.2% of their population was associated with multilevel collapse using DISE. Woodson in 2015 [20] encountered that 15% of patients had single-site ptotic epiglottis using DISE as well as their diagnostic method. Torre et al. [21], in a systematic review done in 2015, found that epiglottis surgery improves outcomes when combined with other procedures in multilevel collapse and CPAP could be ineffective when there is a severe floppy epiglottis collapse. In the majority of cases, epiglottic collapse coexists with obstruction at other sites, whereas isolated epiglottic collapse is observed in significantly fewer patients, ranging from 3.5% to 14.4% [22].

Lan et al. [19] observed that 12.5% of patients had partial anteroposterior epiglottis (AP) collapse, while a larger number (26.6%) had a complete collapse. Similar results were also observed by Ravesloot et al. [23] who, out of 100 consecutive patients undergoing DISE, found that 12% had partial AP collapse and 16% had a complete collapse. In addition, in this study, 2% of patients had a partial lateral collapse and 8% had a complete collapse.

It is important to note that the prevalence of epiglottic collapse was found to be significantly higher when evaluating patients with OSA who have previously failed upper airway surgery, ranging from 44% to 72.9% [24,25].

The aim of this study is to review DISE findings in our tertiary center and classify the different epiglottic collapse patterns (Francia-Lugo classification).

## 2. Materials and Methods

### 2.1. Patients Selection

This study was retrospectively performed in the Instituto de Seguridad y Servicios Sociales de los Trabajadores del Estado (ISSSTE), Clínica Hospital Constitución, Monterrey, Nueva León, México—Sleep Disorders Clinic. All DISE videos performed and data obtained between January 2012 and December 2019 were reviewed by our team.

The inclusion criteria were as follows: (1) adult patients (18 to 64 years old); (2) OSA diagnosed (Apnea-Hypopnea Index—AHI > 10) by a non-attended polysomnography type 3, according to The American Academy of Sleep Medicine (AASM) standard [26]; (3) with no comorbidities or minor metabolic problems (controlled hypertension, body mass index (BMI) < 35); (4) who has poor adaptation (usage of <4 h per night on 70% of nights during a consecutive 30-day period) [27] and/or pursuit an alternative treatment to CPAP therapy; and (5) who has been selected for DISE examination with an epiglottis collapse (VOTE more than 50% in the epiglottis site), either isolated or in a multilevel pattern, were included.

An informed consent was conducted prior to enrollment and authorization was given from the director and main professor of the Sleep Disorders Clinic to review the videos and necessary data in the files.

### 2.2. DISE Procedure

DISE was performed by an otolaryngologist and anesthesia was provided by an anesthesiologist in an ambulatory operating room or in an endoscopic area. We performed a positional exploration in all our patients, starting with supine position. A loading infusion of dexmedetomidine was started at 1 to 1.5 mcg/kg over 10–15 min followed by a maintenance infusion of 1–1.5 mcg/kg/h until completion of the DISE as recently described by Han et al. [28].

Inclusion criteria for DISE examination were as follows: poor adherence to CPAP therapy, defining “adherence” according to US MEDICARE criteria as the use of CPAP at least 4 h each night for 70% of nights during a consecutive 30-day period, during the first 3 months of initial usage [27], and alternative treatment to CPAP therapy decided by the patient and approved by the sleep clinic.

We categorized the obstructive site using *VOTE* classification (*V* velum; *O* oropharynx; *T* tongue base and *E* epiglottis are the sites evaluated). The site, type, and degree of obstruction were recorded.

In this study we focused on patients with more than 50% of epiglottis collapse (defined as complete) and described their visual characteristics.

## 3. Results

We reviewed 634 DISE in which 104 patients (16.4%) were found with epiglottis collapse. We found that 79 patients had primary isolated epiglottis collapse (12.5%) and 25 patients had secondary collapse due to base tongue or lateral pharyngeal walls (3.9%). We describe 4 different types of collapse:

1. Anterior–Posterior (AP) primary collapse with rigid component “trapdoor type” in 50 patients (48%);
2. AP primary collapse with lax component “floppy type” in 14 patients (13.5%);
3. Lateral–Lateral (LL) primary collapse with omega shape component “book type” in 15 patients (14.5%).
4. Secondary collapse due to base tongue or lateral pharyngeal walls collapse in 25 patients (24%).

**Figure 1:**
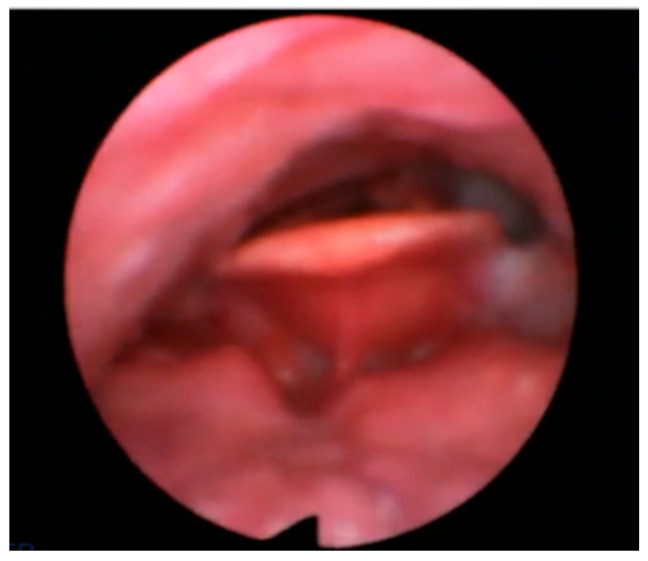
AP primary collapse with rigid component (trapdoor).

**Figure 2:**
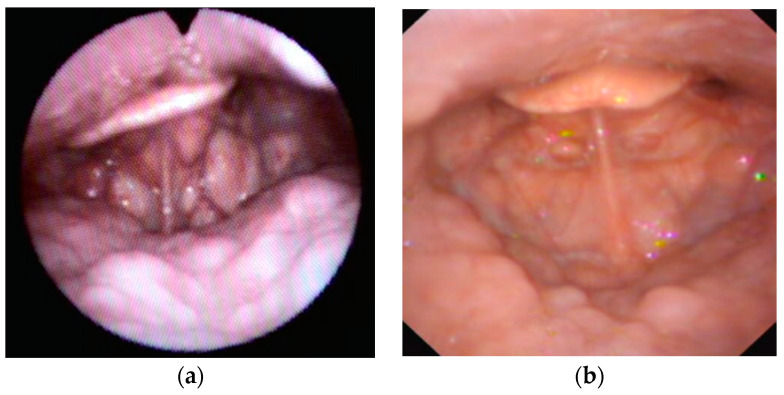
Examples of AP primary collapse with hyperlax epiglottis (floppy).

**Figure 3:**
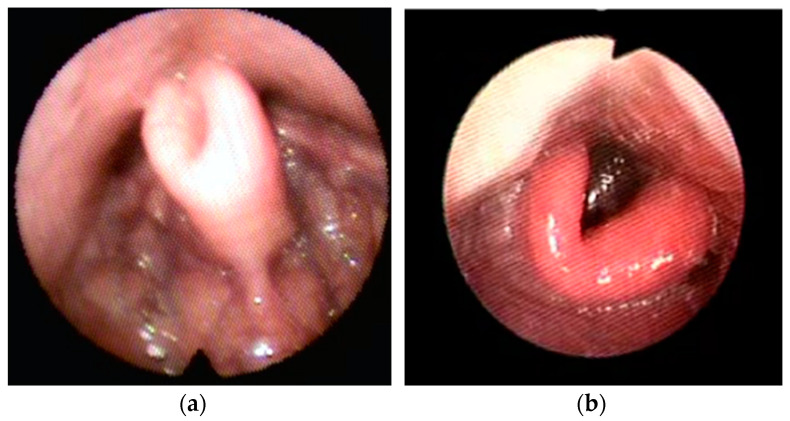
Examples of Lateral– Lateral (LL) primary collapse with omega-shape epiglottis (book type).

**Figure 4:**
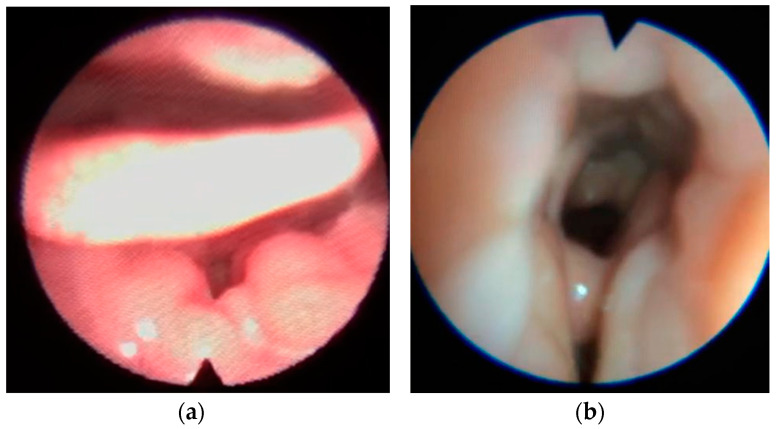
Secondary epiglottic collapse due to tongue base (**a**) or lateral pharyngeal walls collapse (**b**).

Examples of the four types of collapse is shown in Figure 1, Figure 2, Figure 3 and Figure 4.

## 4. Discussion

The epiglottis is an important anatomical structure that was mainly ignored and/or underestimated in the early research in obstructive breathing disorders [18].

Recent studies, have shown that it plays a pivotal role, either on when it is alone or in combination with other pharyngeal structures [29].

Before the advent of DISE, epiglottis collapse was found on the basis of awake examinations. Its prevalence is thought to be 11.5% [18]. DISE has shown that prevalence of epiglottis collapse is variable and tend to be higher than previously described [19,30].

Spinowitz et al. described in a series of 54 patients included in DISE with upper airway resistance syndrome (UARS) non OSA, that 39% had complete epiglottis collapse without mentioning an isolated or multilevel type of obstruction [31].

In our paper we encountered 16.4% of epiglottis collapse, described as primary 12.5% and secondary due to tongue base or lateral walls collapse 3.9%; we included strictly patients with more than 50% of collapse (VOTE classification) and performed the DISE with dexmedetomidine drug, it may play an important fact in the incidence of our findings. In addition, it has also been shown that AP epiglottis collapse is more frequently than lateral epiglottis collapse and is more common in patients who do not respond to upper airway surgery for OSA [32,33].

VOTE classification is one of the most used classification in DISE [16]. Other classifications as NOHL [34] describes the presence of the collapse nor the pattern.

There are a few studies describing epiglottis collapse patterns in OSA patients. Torre et al. in a systematic review done in 2015 described two types of epiglottis collapse: AP and lateral (AP being predominant) [21]. Ravesloot et al. in a study of 100 patients undergoing DISE found that 24% had AP collapse and 10% had lateral collapse, respectively [23]. In 2013, Salama et al. described three types of epiglottis based on their shape, these were flat, curled and omega; they found that the omega shaped epiglottis was associated with the highest AHI, desaturation index, lowest average and minimum oxygen level [35]. In 2020, Vonk et al. found that 16.3% of their patients had floppy epiglottis during DISE and adopting a lateral position had a promising alternative treatment [36].

According to our findings, we summarized the different epiglottic collapses in two major categories named the Francia–Lugo Classification:

**Primary or intrinsic epiglottic collapse (76%):** where the collapse is due to a structural defect of epiglottic. There are three types of primary epiglottic collapse, as follows:

- AP collapse with a rigid component (trapdoor).
- AP collapse with a hyperlax epiglottis (floppy).
- LL collapse with an omega-shaped epiglottis (book type).

**Secondary or extrinsic epiglottic collapse (24%)**: due to compression by other structures (tongue base collapse or lateral pharyngeal walls).

Identification of the epiglottic collapse pattern is crucial in order to solve or improve sleep apnea. The first line of treatment for OSA continues to be CPAP. Actually, previous evidence suggests that CPAP may push the epiglottis further down into the entrance of the larynx, thus being ineffective in treating OSA patients who present with epiglottis collapse. Furthermore, it has been hypothesized that in the presence of a “floppy epiglottis”, the forced pressure generated by CPAP could cause the epiglottis to move backward to the point of complete occlusion at the laryngeal entrance [21,37]. This would result in worsening OSA. In contrast, good results in treating epiglottis collapse has been shown by positional therapy, even more effective than oral appliances in OSA patients [38,39].

Interestingly, OSA patients with epiglottis collapse appear to be thinner than common patients with OSA. HY Kim et al. in a case–control study encountered that patients with epiglottic collapse (AP type) showed significantly lower BMI and obstructive sleep apnea severity (lower AHI, higher lowest saturation percentage) [39].

Another factor that predisposes patients to OSA is craniofacial deformity. Similarly, epiglottis collapse is usually unrelated to this characteristic.

In our experience we have seen more young and slim patients with epiglottic collapse with different degree of OSA. Therefore, it is crucial to look about these kind of collapse in these type of patients [21,39,40].

According to the technique described in the literature, to date, the CO_2_ laser and robotic surgery are used for the reduction of epiglottic.

For the treatment of primary epiglottic collapse confirmed by DISE, Lugo et al. [41] suggested that the surgical procedure should be according to the type and extent of the collapse, which are listed as follows:

- Epiglottic with lateral or book cracking, partial resection superior to 75%.
- Epiglottis with AP collapse “floppy” collapse, partial resection of 50% with epiglottopexy.
- Epiglottis with AP crack rigid collapse “trapdoor”, partial resection less than 50% with or without epiglottopexy.

To date, different surgical approaches have been described with the aim of treating this peculiar region, but some of these are technically complex and associated with complications, such as bleeding, edema, persistent dysphagia or dysgeusia [42,43].

Recently, Salamanca et al. [42] described a new surgical procedure called the “epiglottis stiffening operation” (ESO). It is well tolerated and very effective in the treatment of primary epiglottic collapse especially for “floppy collapse” without altering the fundamental functions of the epiglottis.

Furthermore, in the case of the omega-shaped epiglottis, horizontally extended cauterization leads to a simultaneous epiglottis opening and stiffening.

It is crucial to take into account the functions of the epiglottis. Indeed, the epiglottis is involved in the prevention of food aspiration in two ways: by mechanically closing the laryngeal aditus during swallowing, and by contributing to swallowing through its sensitive receptors distributed on its surface. The main contraindications for the epiglottic surgical procedures are as follows: (1) secondary collapse or extrinsic collapse; (2) concomitant laryngeal pathology with risk of aspiration; (3) inadequate DISE technique; (4) lack of DISE before the surgical procedure. To prevent these unwanted complications, it is important from a technical point of view to leave a 3–4 mm rim of healthy mucosa along the entire profile of the epiglottis [38].

## 5. Conclusions

OSA is commonly presented with multilevel obstructions, and identification of epiglottic collapse may improve the surgical success rate. The Francia–Lugo classification findings reviewed in DISE, assists to understand OSA phenotypes. It could have a major impact on the type of treatment or surgery chosen. Additional research is needed to measure the outcomes of these patients depending on the type of collapse, isolated or multilevel concomitant treatment and the relationship between the clinical characteristics and collapse patterns.

## Data Availability

The data presented in this study are available on request from the corresponding author. The data are not publicly available due to the originality of the work.
